# An evaluation of the impact of a restrictive retail food environment intervention in a rural community pharmacy setting

**DOI:** 10.1186/s12889-016-3281-9

**Published:** 2016-07-16

**Authors:** Leia M. Minaker, Dana Lee Olstad, Graham MacKenzie, Nghia Nguyen, Sunday Azagba, Brian E. Cook, Catherine L. Mah

**Affiliations:** Propel Centre for Population Health Impact, Faculty of Applied Health Sciences, University of Waterloo, 200 University Avenue West, Waterloo, ON N2L 3G1 Canada; Centre for Physical Activity and Nutrition Research, School of Exercise and Nutrition Sciences, Faculty of Health, Deakin University, Melbourne, Australia; Atlantic Pharmasave, 491 Chebucto St, Baddeck, NS B0E 1B0 Canada; Toronto Public Health, 277 Victoria Street, 5th Floor, Toronto, ON M5B 1W2 Canada; Community Health and Humanities, Faculty of Medicine, Memorial University of Newfoundland, 230 Elizabeth Ave, St. John’s, NL A1B 3X9 Canada

## Abstract

**Background:**

Sugar-sweetened beverage consumption is associated with morbidity and mortality. The retail food environment influences food and beverage purchasing and consumption. This study assesses the impact of a community pharmacy’s removal of sweet beverages on overall community sales of carbonated soft drinks (CSD) in a rural setting. We also examined whether the pharmacy intervention affected CSD sales in the town’s other food stores.

**Methods:**

Weekly CSD sales data were acquired from the three food retailers in the town of Baddeck, Nova Scotia (January 1, 2013 to May 8, 2015, *n* = 123 weeks). Autoregressive integrated moving average (ARIMA) analysis was used to analyse the interrupted time series data and estimate the impact of the pharmacy intervention (September 11, 2014) on overall CSD sales at the community level. Data were analysed in 2015.

**Results:**

Before the intervention, the pharmacy accounted for approximately 6 % of CSD sales in the community. After the intervention, declines in total weekly average community CSD sales were not statistically significantly. CSD sales at the other food stores did not increase after the pharmacy intervention.

**Conclusions:**

This study was among the first to examine the impact of a restrictive retail food environment intervention, and found a non-significant decline in CSD sales at the community level. It is the first study to examine a retail food environment intervention in a community pharmacy. Pharmacies may have an important role to play in creating healthy retail food environments.

## Background

Excess sugar consumption has been linked to weight gain, obesity, type 2 diabetes, and cardio-vascular risk factors [1–3]. Globally, sugar-sweetened beverage consumption accounts for approximately 184,000 deaths annually [4]. New WHO guidelines recommend adults and children consume less than 10 % of their total energy intake as free sugars, and encourage a further reduction to below 5 % (about 25 g or 6 teaspoons) for additional health benefits [5]. To contextualize this amount, one 355 mL can of Coca-Cola contains 39 g (about 10 teaspoons of sugar), approximately 8 % of the daily energy requirement for an individual [6].

Food consumption and its downstream effects on health are constrained and embedded within individuals’ social, economic, and physical environments [7–11]. Food environments facilitate access to unhealthy foods by exploiting individuals’ biological, psychological, social, and economic vulnerabilities [12]. Over 70 cents of every household food dollar is spent in stores (as opposed to restaurants) [13]. Retail food environment (RFE) interventions are interventions in food stores and restaurants that aim to support healthier dietary behaviours by improving access to and availability of affordable, healthier food options in the community and consumer nutrition environments [14]. Examples include zoning regulations to restrict fast food outlets from opening in neighbourhoods, [15] ‘healthy corner store’ programs, [16] and point-of-purchase information in grocery stores [17]. Kremers notes that energy-balance related behaviours (such as sugar consumption) can be governed by environmental cues through automatic environment-behavior links rather than through individuals’ conscious processing and deliberate choices [18]. Retail food environment interventions thus aim to shift environmental cues towards health-promoting dietary intake, and away from disease-promoting dietary intake.

Existing RFE interventions have typically been permissive in nature, rather than restrictive. Permissive interventions nudge consumers towards healthier food purchasing by promoting availability of affordable, nutrient-dense foods. Conversely, restrictive interventions aim to decrease availability of calorie-dense, nutrient-poor foods. Restrictive interventions are less well represented in the literature [16, 19].

This study examines an intriguing example of a recent retailer-led restrictive RFE intervention in the province of Nova Scotia, Canada. In September 2014, a pharmacist made national and regional news by removing all sweet beverages from his pharmacy’s shelves [20, 21]. When questioned about his objectives, the pharmacist commented, “It made no sense to me. Just in good conscience, we just couldn’t continue selling” [20]. This retailer-led action constituted an excellent opportunity to conduct a natural experiment to evaluate the impact of a restrictive intervention. The objective of this study was to assess the effect of restricting availability of unhealthy beverage options in the pharmacy on sales of carbonated soft drinks (CSD) at the community level. We also examined whether there was any evidence of “switching” behaviour among stores in the town. That is, did removal of CSD from the pharmacy affect sales of CSD in the other Baddeck food stores? Given that many CSD purchases represent impulsive decisions, [22] we hypothesized that CSD sales would not increase at the other food stores in Baddeck, and that there would be an overall net decline in CSD sales.

## Methods

### Context

Baddeck is a rural small town (population approximately 800 year-round residents) on Cape Breton Island in Nova Scotia. Baddeck is a popular tourist destination in the summer months, and throughout the year functions as a service center for the surrounding, sparsely populated county. The nearest urban municipality is Sydney, Nova Scotia (population 31,597), 80 km away (about an hour’s drive). The community nutrition environment in Baddeck includes twelve restaurants (several of which are only open seasonally), and three food stores including the pharmacy. In September 2014, the owner of the pharmacy pulled all sugar- and artificially-sweetened beverages from his shelves but continued to sell milk and water. The pharmacist’s decision to stop selling these beverages made national and regional news [20, 21, 23].

### Data sources

Weekly sales data on sweet beverages were requested from all three retail food stores in Baddeck. Store 1 and Store 2 consented to provide detailed CSD but not other sweet beverage data, such as juice. Therefore, although the pharmacy ban encompassed all sweet beverages, our study analyzes only sales of CSD (including diet and regular varieties). For the purpose of this study, CSD are considered sweetened drinks that contain carbonated water (including artificially sweetened drinks as well as drinks sweetened with sugar or fruit juice). Weekly sales (in Canadian dollars) of CSD were acquired from the three food stores in Baddeck from January 1, 2013 (the earliest date data were readily available) to May 8, 2015 (the date data were requested) (*n* = 123 weeks). CSD sales peaked in June, July and August, consistent with Canadian data on seasonality of CSD sales [22]. Weeks were categorized in a dichotomous summer peak variable, where weeks in June, July and August were coded as summer peak weeks. Weeks were also categorized as pre-intervention (between January 1, 2013 and September 11, 2014, which was the date that the sweet beverages were pulled from shelves; *n* = 88 weeks) and post-intervention (September 11, 2014 to May 8, 2015; *n* = 35 weeks).

### Statistical analysis

Descriptive statistics were used to examine the mean weekly sales and 95 % confidence interval of the mean for pre-intervention and post-intervention periods, as well as for summer peak weeks and non-peak weeks. Preliminary t-tests were run to determine whether mean weekly sales differed significantly by intervention presence (pre/post) and by peak versus non-peak sale weeks (yes/no).

Although several analytical options exist to analyse time series data, [24] our interrupted time series data were most appropriately analysed using autoregressive integrated moving average (ARIMA) models. These models attempt to account for all aspects of data series autocorrelation, and are appropriate for repeated measures data assessed at equal intervals. We employed a systematic process for each ARIMA model using three standard procedures: model identification, parameter estimation, and diagnostic checking. We fit and compared various ARIMA models, including autoregressive, moving average, or autoregressive moving average models. The simplest model that best described the behaviour of the time series was selected. Differencing (i.e., calculating differences among pairs of observations at some time lag) was used to achieve stationarity. Detailed model specifications are available upon request. Briefly, the time-series data were highly auto-correlated. Data were differenced once to achieve stationarity. Adequacy of all candidate models were assessed visually with autocorrelation function and partial autocorrelation function plots, Ljung-Box chi-square tests for normally distributed white noise residuals, and Q-Q plots and normal distribution histograms of residuals. Finally, Akaike’s information criteria (AIC) and Bayesian information criterion (BIC) were used to establish model fit.

We present two final ARIMA 110 models (for AR = 1, diff = 1 and MA = 0) for the community as a whole, which represent the sum of all three stores’ weekly CSD sales. Final models converged well and were adequate as determined by the diagnostic tests noted above. The first ARIMA model included the variables week number, policy, and summer peak. The second ARIMA model included the variables week number, policy, and seasonality as defined by the ARIMA procedure.

To assess switching behaviour, we also created two ARIMA 110 models (for AR = 1, diff = 1 and MA = 0) for Store 1 and Store 2 individually. If consumers who purchased CSD from the pharmacy switched to Store 1 or Store 2 after the policy to purchase CSD, we would expect to see increases in CSD sales in those stores. Statistical significance was considered *p* < 0.05. Analyses were performed using PROC ARIMA in SAS V.9.3.

Institutional research ethics board approval was not sought for this study as it did not involve data collection from human subjects.

## Results

**Fig. 1:**
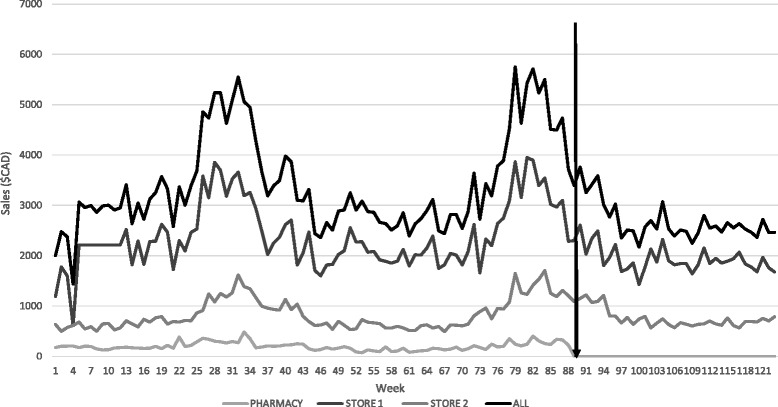
Weekly sales ($CAD) of carbonated soft drinks, Baddeck, Nova Scotia, January 1, 2013 to May 8, 2015. The black arrow represents time of policy introduction. Carbonated soft drinks include diet and regular varieties

Figure 1 shows weekly CSD sales from all three food stores in Baddeck from January 1, 2013 to May 8, 2015. Pre-intervention, the pharmacy accounted for approximately 6 % of total CSD sales ($CAD) in Baddeck. The two peaks in the graph demonstrate two CSD sales peaks in the summer months (June, July and August) in 2013 and 2014. Table 1 shows descriptive statistics of weekly CSD sales from each store. For all stores, weekly CSD sales were significantly lower in non-peak weeks relative to summer peak weeks.

**Table 1:** Summary of weekly sales ($CAD) of carbonated soft drinks in Baddeck, Nova Scotia, January 1, 2013 to May 8, 2015

	Weekly Sales			
Store	Pre- policy mean (95 % CI)	Post- policy mean (95 % CI)	Non-peak months mean (95 % CI)	Summer peak months mean (95 % CI)
Pharmacy	201.20 (184.50, 217.80)	0.00	106.4 (88.30, 124.50)	283.80 (254.40, 313.30)
Store 1	2401.40 (2263.70, 2539.1)	1938.40 (1851.60, 2025.10)	2025.50 (1959.60, 2091.30)	3180.70 (2971.80, 3389.60)
Store 2	843.80 (769.80, 899.70)	761.50 (696.80, 826.30)	712.60 (677.50, 747.60)	1192.00 (1080.60, 1303.50)
All stores	3437.40 (3231.6, 3643.1)	2699.90 (2568.40, 2831.30)	2844.50 (2756.30, 2932.60)	4656.60 (4344.60, 4968.50)

Carbonated soft drinks include diet and regular varieties

Table 2 shows the results from the two final ARIMA models. Baseline weekly average CSD sales were $3105.10. Summer peak was significantly associated with higher weekly CSD sales at the community level. Model 1 illustrates that after controlling for summer peak, weekly CSD sales were $353.60 (11.4 %) lower in the community as a whole in the post-intervention period (*p* = 0.39). Model 2 shows that after controlling for model-specified seasonality, weekly CSD sales were $663.10 (21.4 %) lower at the community level, post-intervention (*p* = 0.13).

**Table 2:** Results from ARIMA models showing estimates of impacts of policy and summer peak or seasonality on weekly sales ($CAD) of carbonated soft drinks in Baddeck, Nova Scotia, January 1, 2013 to May 8, 2015

Stores	Model 1 (with summer peak)		Model 2 (with ARIMA-specified seasonality)	
	Estimate (SE)	*p*-value	Estimate (SE)	*p*-value
All stores				
Baseline level	3105.1 (192.6)	<0.0001	3105.1 (192.6)	<0.0001
Policy	−353.60 (413.00)	0.39	−663.10 (439.50)	0.13
Summer peak	533.70 (205.50)	0.01		

Carbonated soft drinks include diet and regular varieties

ARIMA 110 model estimates

Table 3 shows the results from two ARIMA models (using Store 1 data and then Store 2 data) illustrating potential switching behaviours. No significant increase in CSD sales was observed in either of the stores.

**Table 3:** Results from ARIMA models showing estimates of impacts of policy on weekly sales ($CAD) of carbonated soft drinks in Store 1 and Store 2 (non-intervention retailers), controlling for summer peak in Baddeck, Nova Scotia, January 1, 2013 to May 8, 2015

Stores	Store 1		Store 2	
	Estimate (SE)	*p*-value	Estimate (SE)	*p*-value
Policy	21.40 (343.90)	0.95	−115.80 (133.20)	0.39
Summer peak	488.40 (205.50)	0.005	46.00 (65.00)	0.49

## Discussion

This study examined the impact of a restrictive RFE intervention on weekly CSD sales at the community level. The analysis contributes to the limited literature on impact of restrictive interventions. We found that after controlling for seasonal variations in CSD sales, a restrictive retailer-led intervention banning sales of CSD in a community pharmacy was associated with a non-significant decline in sales of CSD at the community level in Baddeck, Nova Scotia. Another key finding was that there was no evidence of “switching behaviour”, that is, consumers did not buy more CSD from Store 1 or Store 2 after the pharmacy stopped selling CSD. This finding is meaningful from a population health intervention standpoint in particular, as it indicates that the pharmacy, rather than being a purposeful consumer destination for CSD purchasing, may actually have acted mainly as a source of impulse CSD purchasing within the community nutrition environment, as has been suggested previously [22].

Evidence on the impact of RFE interventions on diets and health has typically been generated by evaluations of permissive rather than restrictive interventions [16, 19]. Recent literature suggests mixed results of permissive interventions on healthy food purchasing [16, 25] and/or dietary intake [16, 17, 26]. While permissive interventions increase access to and availability of healthful options, such interventions may not be optimally effective without compatible efforts to restrict the availability of less healthy choices—producing an overall net food environment shift. This study contributes to the literature on restrictive RFE interventions, and finds a non-statistically significant impact of a pharmacy intervention in a small rural community.

Further research is required to analyze the myriad retail settings in which food and beverages are now sold. Industry analyses suggest that revenues of large chain pharmacies are split evenly between front-of store sales (e.g., foods and beverages, household items) and prescriptions, although smaller and independent pharmacies still generate most of their revenue through prescription sales [27]. The relatively minor proportion of revenue derived from the sale of sweet beverages at the study pharmacy facilitated the pharmacist’s decision to remove them without risking significant declines in revenues. Pharmacies may have an improved capacity to accommodate restrictive interventions as part of their business model, and may therefore be ideal settings in which to test these interventions. To the extent that such interventions can be established as profitable—or at least not a major financial risk—it will increase the potential for scaling up implementation among other small, independent food retailers.

Community pharmacies are health care settings that have been identified as an important health promotion setting given their geographic accessibility, diverse customer base, and the high level of public confidence in pharmacists [28–31]. Programs that engage pharmacies as health promotion settings typically focus on community pharmacists’ potential role as nutrition counsellors [29–32]. However, the dual roles of many pharmacists, first as health care professionals and second as business owners, can sometimes conflict. For example, the literature suggests that consumers [30] and nutritionists [31] perceive pharmacists who sell infant formula and bottles as having a conflict of interest in promoting breast-feeding. Consumers also perceive financial conflicts of interests when pharmacists recommend specific weight loss products they sell [29, 32]. Indeed, for community pharmacists, “both patient care and viability of the business need to be maintained” [32]. Therefore, interventions to promote healthy diets in pharmacies must also be economically feasible.

Financial conflicts of interest related to products sold at pharmacies are not new. Tobacco sales in pharmacies have spurred much debate over pharmacists’ role as health care professionals, given that tobacco use continues to be the leading cause of preventable death in the world [33]. Nine of 10 Canadian provinces prohibit tobacco sales in pharmacies [34]. While sugar-sweetened beverages are not typically regarded as being as harmful as tobacco products, sugar-sweetened beverage consumption is harmful to health [1, 2]. When CVS/Caremark announced it would stop selling tobacco in 2014, it did so based on carefully considered costs, including the normative and social costs of selling tobacco, which were high [34]. Future research should continue to monitor public opinion of sugar-sweetened beverages as a way of understanding normative and social risks for pharmacies that sell sugar sweetened beverages.

Several limitations of this study should be noted. First, we were unable to examine consumption phenomena at the individual/household level such as substitution effects. That is, we were unable to determine whether consumers who purchased less CSD ultimately increased their purchase of other types of beverages, including more or less nutritious beverages. Second, this analysis was also unable to distinguish between sales of diet and regular CSD. It is therefore unclear what proportion of the decline in sales of CSD was due to a reduction in sales of diet CSD. Industry analysis suggests that diet soft drinks have historically accounted for a small proportion of beverage sales [35]. It is therefore likely that observed declines were largely attributable to reductions in sales of sugar-sweetened CSD. Third, there was no control community in this natural experiment. The secular decline cited in industry documentation has not been as large as the declines observed in the current study, with total CSD sales volume declining approximately 3 % over same time period in the study, since 2013 [35]. Further, most food environment intervention studies have used an uncontrolled pre-post design since the interventions are often outside of the researchers’ control [19]. Finally, the sales data we obtained from the retailers did not include quantity of volume of CSD. This data limitation means that we were not able to detect the actual volume of CSD purchased. Considering tiered pricing strategies (i.e., CSD in larger-volume bottles cost less per ounce than CSD in smaller-volume bottles), if consumers switched to purchasing larger CSD bottles, our data would not reflect this phenomenon.

It is possible that people living in Baddeck would drive outside of their town to procure CSD. However, given that the nearest larger center is about an hour’s drive away, and given that CSD were still available in both Store 1 and Store 2, it is unlikely that there was a change in out-of-town CSD procurement. Finally, it is likely that the lack of statistical significance accompanying the estimates was caused by the relatively short follow-up time (*n* = 35 weeks) and by the large standard errors reflected in large weekly variations in CSD sales. In addition, the relatively short follow-up time precluded an analysis of CSD during a summer peak in which the policy was in place, when a different type of consumer (namely tourists) would have comprised a greater proportion of people purchasing CSD.

Despite these limitations, this study was among the first to examine the impact of a restrictive RFE intervention on CSD sales at the community level. An important strength of this study is that objective weekly CSD sales data were obtained from all retail stores selling beverages in the study community over a 123 week period. The strength of the ARIMA model in addressing both autocorrelation in the data as well as seasonal variation in weekly CSD sales is also notable.

## Conclusions

This is a promising area of inquiry that would benefit from research directions that engage complementary disciplinary expertise. For example, determining the level of public support for the pharmacy intervention would be a first step to understanding the feasibility and acceptability of this type of intervention. The financial impact of voluntary prohibitions on the sale of sugar-sweetened beverages in pharmacies should also be examined. Finally, future research should further examine the extent to which declines in the sales of CSD translate into reduced CSD consumption at the individual/household level.

## Abbreviations

RFE, ARIMA
